# Redetermination of 3-(ammonio­meth­yl)pyridinium dichloride

**DOI:** 10.1107/S1600536809025859

**Published:** 2009-07-11

**Authors:** Wen-Xian Liang, Gang Wang, Zhi-Rong Qu

**Affiliations:** a Ordered Matter Science Research Center, College of Chemistry and Chemical Engineering, Southeast University, Nanjing 210096, People’s Republic of China

## Abstract

The crystal structure of the title compound, C_6_H_10_N_2_^2+^·2Cl^−^, has been reported previously in the non-standard setting *P*2_1_/*a* [Genet (1965 ▶). *Bull. Soc. Fr. Miner. Crist.* **88**, 463–470], with an *R* value of 0.16. The current redetermination improves significantly the precision of the geometric parameters. In the crystal packing, cations and anions are linked by inter­molecular N—H⋯Cl and C—H⋯Cl hydrogen bonds into a three-dimensional network.

## Related literature

For related structures, see: Genet (1965 ▶); Chtioui & Jouini (2004 ▶); Long *et al.* (1997 ▶).
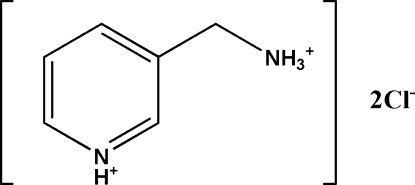


## Experimental

### 

#### Crystal data

C_6_H_10_N_2_^2+^·2Cl^−^
*M* *_r_* = 181.06
Monoclinic, 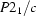

*a* = 4.5874 (9) Å
*b* = 12.650 (3) Å
*c* = 14.814 (3) Å
β = 93.61 (3)°
*V* = 857.9 (3) Å^3^
*Z* = 4
Mo *K*α radiation
μ = 0.69 mm^−1^
*T* = 293 K
0.50 × 0.45 × 0.15 mm

#### Data collection

Rigaku SCXmini diffractometer
Absorption correction: multi-scan (*CrystalClear*; Rigaku, 2005 ▶) *T*_min_ = 0.720, *T*_max_ = 0.909
8831 measured reflections
1961 independent reflections
1684 reflections with *I* > 2σ(*I*)
*R*_int_ = 0.035

#### Refinement

*R*[*F* ^2^ > 2σ(*F* ^2^)] = 0.033
*wR*(*F* ^2^) = 0.076
*S* = 1.08
1961 reflections
92 parameters
H-atom parameters constrained
Δρ_max_ = 0.21 e Å^−3^
Δρ_min_ = −0.19 e Å^−3^

### 

Data collection: *CrystalClear* (Rigaku, 2005 ▶); cell refinement: *CrystalClear*; data reduction: *CrystalClear*; program(s) used to solve structure: *SHELXS97* (Sheldrick, 2008 ▶); program(s) used to refine structure: *SHELXL97* (Sheldrick, 2008 ▶); molecular graphics: *SHELXTL* (Sheldrick, 2008 ▶); software used to prepare material for publication: *PRPKAPPA* (Ferguson, 1999 ▶).

## Supplementary Material

Crystal structure: contains datablocks I, global. DOI: 10.1107/S1600536809025859/rz2346sup1.cif

Structure factors: contains datablocks I. DOI: 10.1107/S1600536809025859/rz2346Isup2.hkl

Additional supplementary materials:  crystallographic information; 3D view; checkCIF report

## Figures and Tables

**Table 1:** Hydrogen-bond geometry (Å, °)

*D*—H⋯*A*	*D*—H	H⋯*A*	*D*⋯*A*	*D*—H⋯*A*
N1—H1*A*⋯Cl2^i^	0.89	2.35	3.1914 (16)	157
N1—H1*B*⋯Cl2^ii^	0.89	2.27	3.1206 (16)	159
N1—H1*C*⋯Cl1^iii^	0.89	2.28	3.1622 (16)	170
N2—H2⋯Cl1^iv^	0.86	2.25	3.0520 (16)	154
C3—H3⋯Cl2^i^	0.93	2.77	3.606 (2)	150
C6—H6*A*⋯Cl1^v^	0.97	2.74	3.676 (2)	163
C6—H6*B*⋯Cl2^vi^	0.97	2.82	3.700 (2)	152

Symmetry codes: (i) 
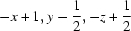
; (ii) 
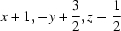
; (iii) 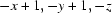; (iv) 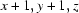; (v) 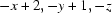; (vi) 
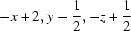
.

**Fig. 1.**
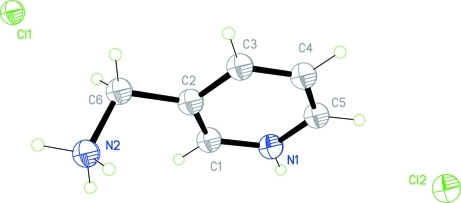
The molecular structure of the title compound, with displacement ellipsoids drawn at the 30% probability level.

**Fig. 2.**
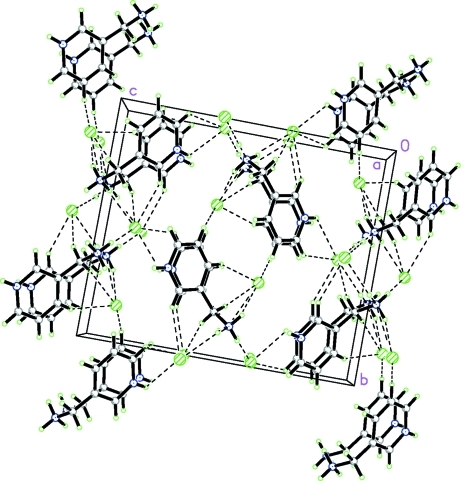
Packing diagram of the title compound viewed along the a axis>. Hydrogen bonds are shown as dashed lines.

## supplementary crystallographic information

### Comment

Pyridin-3-ylmethanamine is a important ligand used in coordination chemistry. Recently, there has been an increased interest in the properties of layer perovskite structures because of their applications in high temperature superconductivity. Two general classes of *M*(II) halide layer perovskite structures exist, the ammoniummethylprididine series (Chtioui & Jouini, 2004) and the ammoniummethylprididinium series (Long *et al.*, 1997). In the latter series, the asymmetrical dication bridges between layers, with both the NH_3_^+^ group and the pyridinium N—H group hydrogen bonding to the halide ions in the layer. The cation-layer interactions involve an ammonium group that hydrogen bonds to the perovskite layer (Long *et al.*, 1997). We report herein the crystal structure of the title compound, which was prepared by the reaction of pyridin-3-ylmethanamine and hydrochloric acid. Its crystal structure has been reported previously in the non standard setting *P*2_1_/a (Genet, 1965), with an R value of 0.16. The current redetermination improves significantly the precision of the geometric parameters.

The asymmetric unit of the title compound (Fig. 1) consists of a two independent chloride anions and a 3-(ammoniomethyl)pyridinium dication. In the cation, the plane through the C2/C6/N2 atoms is tilted by 68.13 (14)° with respect to the pyridine ring. In the crystal packing (Fig. 2), intermolecular N—H···Cl and C—H···Cl hydrogen bonds (Table 1) connect neighbouring cations and anions into a three-dimensional network.

### Experimental

A mixture of (pyridin-3-yl)methanamine (0.1 mol, 0.108 g) and HCl (0.2 mol, 0.73 g) were dissolved in water (10 ml). Colourless single crystals of the title compound suitable for X-ray analysis were obtained on slow evaporation of the solvent over a period of 48 h.

### Refinement

Positional parameters of all the H atoms were calculated geometrically and were allowed to ride on their parent atoms, with C—H = 0.93-0.97 Å, N—H = 0.86-0.89 Å, and with *U*_iso_(H) = 1.2 *U*_eq_(C, N).

### Crystal data

**Table:**

C_6_H_10_N_2_^2+^·2Cl^−^	*F*(000) = 376
*M**_r_* = 181.06	*D*_x_ = 1.402 Mg m^−^^3^
Monoclinic, *P*2_1_/*c*	Mo *K*α radiation, λ = 0.71073 Å
Hall symbol: -P 2ybc	Cell parameters from 8048 reflections
*a* = 4.5874 (9) Å	θ = 3.1–27.5°
*b* = 12.650 (3) Å	µ = 0.69 mm^−^^1^
*c* = 14.814 (3) Å	*T* = 293 K
β = 93.61 (3)°	Prism, colourless
*V* = 857.9 (3) Å^3^	0.50 × 0.45 × 0.15 mm
*Z* = 4	

### Data collection

**Table:**

Rigaku SCXmini diffractometer	1961 independent reflections
Radiation source: fine-focus sealed tube	1684 reflections with *I* > 2σ(*I*)
graphite	*R*_int_ = 0.035
Detector resolution: 13.662 pixels mm^-1^	θ_max_ = 27.5°, θ_min_ = 3.2°
CCD_Profile_fitting scans	*h* = −5→5
Absorption correction: multi-scan (*CrystalClear*; Rigaku, 2005)	*k* = −16→16
*T*_min_ = 0.720, *T*_max_ = 0.909	*l* = −19→19
8831 measured reflections	

### Refinement

**Table:**

Refinement on *F*^2^	Primary atom site location: structure-invariant direct methods
Least-squares matrix: full	Secondary atom site location: difference Fourier map
*R*[*F*^2^ > 2σ(*F*^2^)] = 0.033	Hydrogen site location: inferred from neighbouring sites
*wR*(*F*^2^) = 0.076	H-atom parameters constrained
*S* = 1.08	*w* = 1/[σ^2^(*F*_o_^2^) + (0.0293*P*)^2^ + 0.274*P*] where *P* = (*F*_o_^2^ + 2*F*_c_^2^)/3
1961 reflections	(Δ/σ)_max_ = 0.001
92 parameters	Δρ_max_ = 0.21 e Å^−^^3^
0 restraints	Δρ_min_ = −0.19 e Å^−^^3^

### Special details

**Table:**

Geometry. All e.s.d.'s (except the e.s.d. in the dihedral angle between two l.s. planes) are estimated using the full covariance matrix. The cell e.s.d.'s are taken into account individually in the estimation of e.s.d.'s in distances, angles and torsion angles; correlations between e.s.d.'s in cell parameters are only used when they are defined by crystal symmetry. An approximate (isotropic) treatment of cell e.s.d.'s is used for estimating e.s.d.'s involving l.s. planes.
Refinement. Refinement of *F*^2^ against ALL reflections. The weighted *R*-factor *wR* and goodness of fit *S* are based on *F*^2^, conventional *R*-factors *R* are based on *F*, with *F* set to zero for negative *F*^2^. The threshold expression of *F*^2^ > σ(*F*^2^) is used only for calculating *R*-factors(gt) *etc*. and is not relevant to the choice of reflections for refinement. *R*-factors based on *F*^2^ are statistically about twice as large as those based on *F*, and *R*- factors based on ALL data will be even larger.

### Fractional atomic coordinates and isotropic or equivalent isotropic displacement parameters (Å^2^)

**Table:**

	*x*	*y*	*z*	*U*_iso_*/*U*_eq_	
Cl2	0.34023 (9)	0.98847 (3)	0.37672 (3)	0.03348 (13)	
N1	0.9075 (3)	0.64924 (10)	−0.01920 (10)	0.0333 (3)	
H1A	0.7885	0.6098	0.0120	0.050*	
H1B	0.9905	0.6091	−0.0598	0.050*	
H1C	0.8058	0.7007	−0.0474	0.050*	
C6	1.1364 (4)	0.69588 (14)	0.04332 (13)	0.0365 (4)	
H6A	1.2888	0.7253	0.0084	0.044*	
H6B	1.2231	0.6405	0.0815	0.044*	
C1	1.1087 (4)	0.88417 (13)	0.08977 (12)	0.0337 (4)	
H1	1.2349	0.9000	0.0449	0.040*	
C5	0.8274 (4)	0.94441 (14)	0.20641 (12)	0.0370 (4)	
H5	0.7619	1.0005	0.2403	0.044*	
C2	1.0214 (3)	0.78119 (13)	0.10227 (11)	0.0293 (4)	
C4	0.7351 (4)	0.84350 (14)	0.22213 (12)	0.0371 (4)	
H4	0.6060	0.8304	0.2668	0.045*	
C3	0.8357 (4)	0.76140 (14)	0.17102 (12)	0.0349 (4)	
H3	0.7788	0.6924	0.1826	0.042*	
Cl1	0.40819 (11)	0.14752 (3)	0.10233 (3)	0.04427 (15)	
N2	1.0124 (3)	0.96146 (11)	0.14194 (10)	0.0367 (4)	
H2	1.0724	1.0249	0.1336	0.044*	

### Atomic displacement parameters (Å^2^)

**Table:**

	*U*^11^	*U*^22^	*U*^33^	*U*^12^	*U*^13^	*U*^23^
Cl2	0.0384 (2)	0.0291 (2)	0.0340 (2)	−0.00167 (16)	0.01032 (17)	−0.00133 (16)
N1	0.0389 (8)	0.0256 (7)	0.0362 (8)	0.0047 (6)	0.0099 (6)	−0.0021 (6)
C6	0.0310 (9)	0.0315 (9)	0.0479 (11)	0.0040 (7)	0.0086 (8)	−0.0028 (8)
C1	0.0347 (9)	0.0314 (9)	0.0352 (9)	0.0001 (7)	0.0041 (7)	0.0029 (7)
C5	0.0440 (10)	0.0354 (10)	0.0311 (9)	0.0044 (8)	−0.0011 (8)	−0.0071 (7)
C2	0.0260 (8)	0.0284 (8)	0.0331 (9)	0.0020 (6)	−0.0008 (7)	−0.0010 (7)
C4	0.0406 (10)	0.0407 (10)	0.0306 (9)	−0.0030 (8)	0.0064 (8)	−0.0032 (7)
C3	0.0390 (10)	0.0297 (9)	0.0363 (10)	−0.0038 (7)	0.0044 (8)	0.0005 (7)
Cl1	0.0486 (3)	0.0305 (2)	0.0547 (3)	0.00301 (18)	0.0108 (2)	0.00943 (19)
N2	0.0462 (9)	0.0237 (7)	0.0397 (9)	−0.0029 (6)	−0.0014 (7)	0.0013 (6)

### Geometric parameters (Å, °)

**Table:**

N1—C6	1.478 (2)	C1—H1	0.9300
N1—H1A	0.8900	C5—N2	1.334 (2)
N1—H1B	0.8900	C5—C4	1.369 (3)
N1—H1C	0.8900	C5—H5	0.9300
C6—C2	1.504 (2)	C2—C3	1.391 (2)
C6—H6A	0.9700	C4—C3	1.382 (2)
C6—H6B	0.9700	C4—H4	0.9300
C1—N2	1.338 (2)	C3—H3	0.9300
C1—C2	1.379 (2)	N2—H2	0.8600
			
C6—N1—H1A	109.5	N2—C5—C4	119.33 (16)
C6—N1—H1B	109.5	N2—C5—H5	120.3
H1A—N1—H1B	109.5	C4—C5—H5	120.3
C6—N1—H1C	109.5	C1—C2—C3	117.69 (16)
H1A—N1—H1C	109.5	C1—C2—C6	118.97 (16)
H1B—N1—H1C	109.5	C3—C2—C6	123.31 (15)
N1—C6—C2	112.88 (13)	C5—C4—C3	119.34 (17)
N1—C6—H6A	109.0	C5—C4—H4	120.3
C2—C6—H6A	109.0	C3—C4—H4	120.3
N1—C6—H6B	109.0	C4—C3—C2	120.47 (16)
C2—C6—H6B	109.0	C4—C3—H3	119.8
H6A—C6—H6B	107.8	C2—C3—H3	119.8
N2—C1—C2	120.23 (16)	C5—N2—C1	122.89 (15)
N2—C1—H1	119.9	C5—N2—H2	118.6
C2—C1—H1	119.9	C1—N2—H2	118.6

### Hydrogen-bond geometry (Å, °)

**Table:**

*D*—H···*A*	*D*—H	H···*A*	*D*···*A*	*D*—H···*A*
N1—H1A···Cl2^i^	0.89	2.35	3.1914 (16)	157
N1—H1B···Cl2^ii^	0.89	2.27	3.1206 (16)	159
N1—H1C···Cl1^iii^	0.89	2.28	3.1622 (16)	170
N2—H2···Cl1^iv^	0.86	2.25	3.0520 (16)	154
C3—H3···Cl2^i^	0.93	2.77	3.606 (2)	150
C6—H6A···Cl1^v^	0.97	2.74	3.676 (2)	163
C6—H6B···Cl2^vi^	0.97	2.82	3.700 (2)	152

Symmetry codes: (i) −*x*+1, *y*−1/2, −*z*+1/2; (ii) *x*+1, −*y*+3/2, *z*−1/2; (iii) −*x*+1, −*y*+1, −*z*; (iv) *x*+1, *y*+1, *z*; (v) −*x*+2, −*y*+1, −*z*; (vi) −*x*+2, *y*−1/2, −*z*+1/2.
